# Supplementary material for: Transcriptomic and Metabolomic Analysis of a Pseudomonas-Resistant versus a Susceptible Arabidopsis Accession
Source: Int J Mol Sci. 2022 Oct 11;23(20):12087. doi: 10.3390/ijms232012087 (PMC9603445; doi:10.3390/ijms232012087)
Supplement: Supplementary file 1 [file ijms-23-12087-s001.zip › Orf&Tenenboim_IJMS2022_Pst_supplement_captions.pdf]

Supplementary Tables Captions

Supplementary Table S1: Gene expression in Col-0 and C24 after Pst infection: Plants were either sprayed with Pst (Pst infection) or $MgCl_2$ solution (mock treatment) and samples taken after 2h, 4h, 8h, 16, 24h, and 48h. Gray underlay = significant at specific time point, bold text = significant in interval (Interval 1 = 2h, 4h, 8h; Interval 2 = 16h, 24h, 48h).

Supplementary Table S2: Primary metabolite profiles in Col-0 and C24 after Pst infection: Columns A and B contain metabolite name and the corresponding analyte (GMD). Averages and standard error values of all conditions can be found in column C to AX, 5–7 independent replicates were measured per sample. Significance of changes in metabolite levels between Pst infected and mock treated plants was assessed using Student`s T-Test, performed in R (Columns AY to BJ). Values $P< 0.05$ are highlighted in red.

Supplementary Table S3: Secondary metabolite profiles in Col-0 and C24 after Pst infection: Averages and standard error values of all conditions can be found in column B to AW, 5–7 independent replicates were measured per sample. Significance of changes in metabolite levels between Pst infected and mock treated plants was assessed using Student`s T-Test. Values $P< 0.05$ are highlighted in red.

Supplementary Table S4: Comparison of basal gene expression in Col-0 and C24: Sheet 1 contains all differentially expressed genes (DEGs) between Col-0 and C24. Blue = genes that show higher expression in Col-0, red = genes that show higher expression in C24. Sheets 2-4 contain GO enrichment analyses for all DEGs (Sheet 2), DEGs in Col-0 (Sheet 3), and DEGs in C24 (Sheet 4).

Supplementary Table S5: Overlap of DEG's between soil- and axenic cultures of Arabidopsis: For each DEG (between Col-0 and C24) a difference between its expression in both growth conditions is presented. Two thresholds of interest were applied: fold change > ±1.5 (log2 = 0.585); fold change > ±2 (log2 = 1).

Supplementary Table S6: Shared differentially expressed genes related to defense responses in Col-0 and C24 after Pst infection: Gray underlay = significant at specific time point, bold text = significant in interval (Interval 1 = 2h, 4h, 8h; Interval 2 = 16h, 24h, 48h. Columns BC to BH contain gene ontology IDs and terms. Only genes that belong to GO biological process terms containing "defense ", "systemic", "jasmonic", "ethylene", or "salicylic", and that were significant in both accessions were selected. Every sheet corresponds to one time point.

Supplementary Table S7: Col-0 unique differentially expressed genes related to defense responses after Pst infection: Gray underlay = significant at specific time point, bold text = significant in interval (Interval 1 = 2h, 4h, 8h; Interval 2 = 16h, 24h, 48h). Only genes that belong to GO biological process terms containing "defense ", "systemic", "jasmonic", "ethylene", and "salicylic", and that were significant in Col-0 but not C24 were selected. Every sheet corresponds to one time point.

Supplementary Table S8: C24 unique differentially expressed genes related to defense responses after Pst infection: Gray underlay = significant at specific time point, bold text = significant in interval (Interval 1 = 2h, 4h, 8h; Interval 2 = 16h, 24h, 48h). Only genes that belong to GO biological process terms containing "defense ", "systemic", "jasmonic", "ethylene", and "salicylic", and that were significant in C24 but not Col-0 were selected. Every sheet corresponds to one time point.

Supplementary Table S9: Metabolite Reporting Checklist. The analysis method and processing reproducibility data.

Supplementary Table S10: Metabolite documentation. Documentation of analytes in GC-MS and LC-MS analysis of samples.

Figures

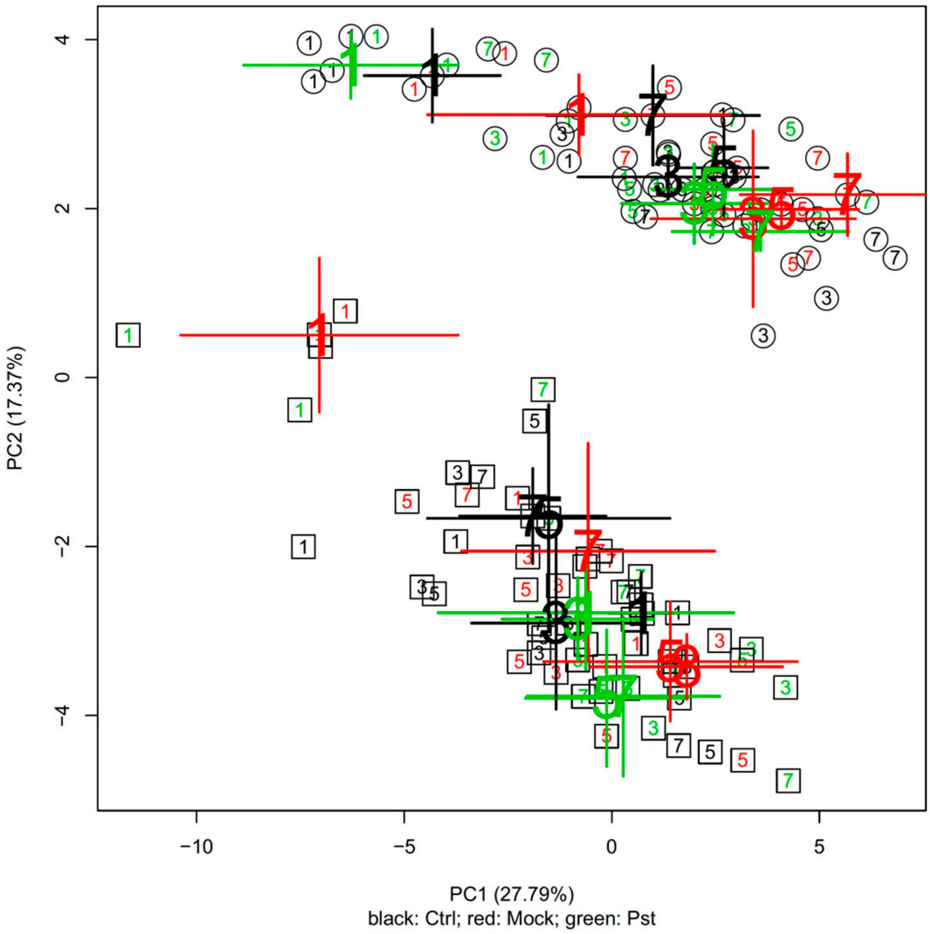

Supplementary Figure 1: Influence of syringe infiltration on metabolite profiles of Col-0 and C24: Primary component analysis of metabolomic profiles of two biological replicate series of plants. Numbers represent time point after infiltration. Color scheme: black, control non-infiltrated plants; red, mock infiltrated plants, green, Pst solution infiltrated plants.

A

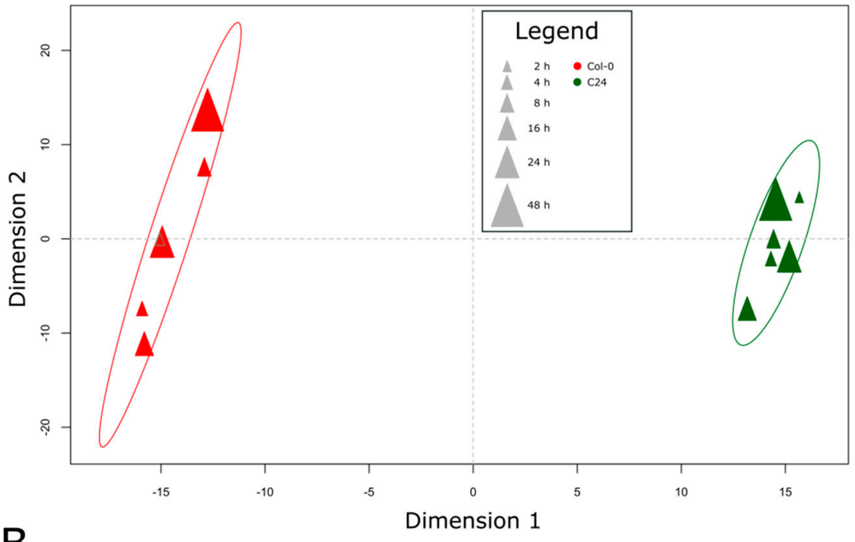

B

1	**pol_neg_18906**	C00007231: CDP-cholineC00307: CDP-choline, Cytidine 5'-diphosphocholine
2	**pol_neg_10369**	C00007352: 7−(Methylsulfinyl)heptyl glucosinolate;Glucoibarin
3	**pol_pos_4535**	C00835: Sepiapterin| C03684: 6−(1,2−Dioxopropyl)−5,6,7,8−tetrahydropterin, 6−Pyruvoyltetrahydropterin | C06313: 2−Amino−6−(1,2−dihydroxypropyl)−4(1H)−pteridinone, Biopterin
4	**pol_neg_10772**	C00007355: 8−Methylsulfinyloctyl glucosinolate;GlucohirsutinC17271: 8−Methylsulfinyloctyl glucosinolate, Glucohirsutin
5	**pol_neg_6916**	C00001488: Sinigrin;2−Phenylethyl glucosinolate;Allyl glucosinolateC08427: 2−Propenyl glucosinolate
6	**pol_neg_9951**	C00007348: 6−Methylsulfinylhexyl glucosinolate;Glucohesperin | C00007813: 3−Hydroxy−6−(methylthio)hexyl glucosinolate
7	**pol_pos_4875**	C06470: 3−O−alpha−Carboxyethyl−D−glucosamine, Muramic acid
8	**pol_pos_9847**	D01508: Phenylacetylglycine dimethylamide (JAN) | C12958: Phenylacetylglycine dimethylamide
9	**pol_pos_15090**	C00006832: Cyanidin3−[6−(6−p−coumarylglucosyl)−2−xylosylgalactoside] | C00006836: Cyanidin 3−(6''−(E)−p−coumarylsambubioside)−5−glucoside | C00014780: Cyanidin 3−(6''−(Z)−p−coumarylsambubioside)−5−glucoside
10	**pol_pos_15615**	C00007355

Supplementary Figure 2: PLS-DA of Col-0 and C24 mock treated plants based on secondary metabolite profiles: Samples in the plot represent averaged profiles of annotated metabolites in 5-7 independent replicates. **A)** Scores plot **B)** List of the top ten metabolite loadings, left column = loadings ranking, middle column = peak identifier, right column = putative metabolite identity based on an in-house database of standard compounds and on KNApSAcK and PubChem.

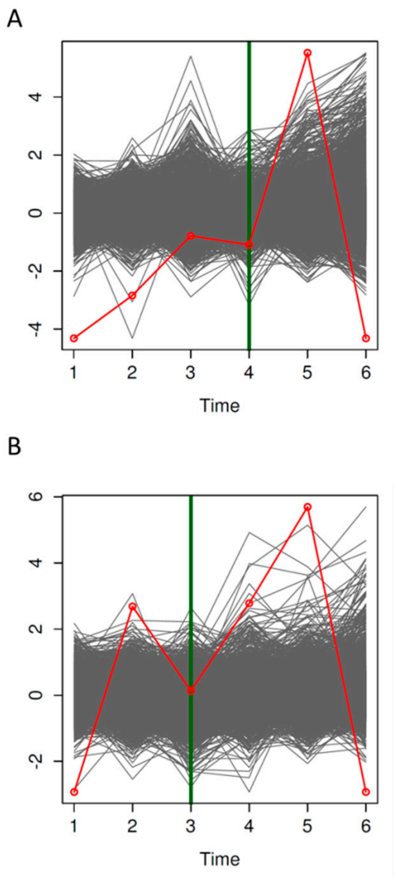

Supplementary Figure 3: Segmentation analysis. Panel A, Col-0; panel B C24. Regression-based formalization of the segmenting multivariate time-series data based on temporal changes in covariance structure. Breakpoints were defined as time points for which the explanatory power of the precedent time points are negligible; likewise, breakpoints have small explanatory contribution for their following sequence of time points. The green line show the obtained breakpoint. The red dots, connected by a red line, represent the column-averages of the absolute values of the regression coefficients. The x-axis represents time points and the y-axis shows the log fold changes.
