# Supplementary figures and images for: Transcriptomic and Metabolomic Analysis of a Pseudomonas-Resistant versus a Susceptible Arabidopsis Accession
Source: Int J Mol Sci. 2022 Oct 11;23(20):12087. doi: 10.3390/ijms232012087 (PMC9603445; doi:10.3390/ijms232012087)

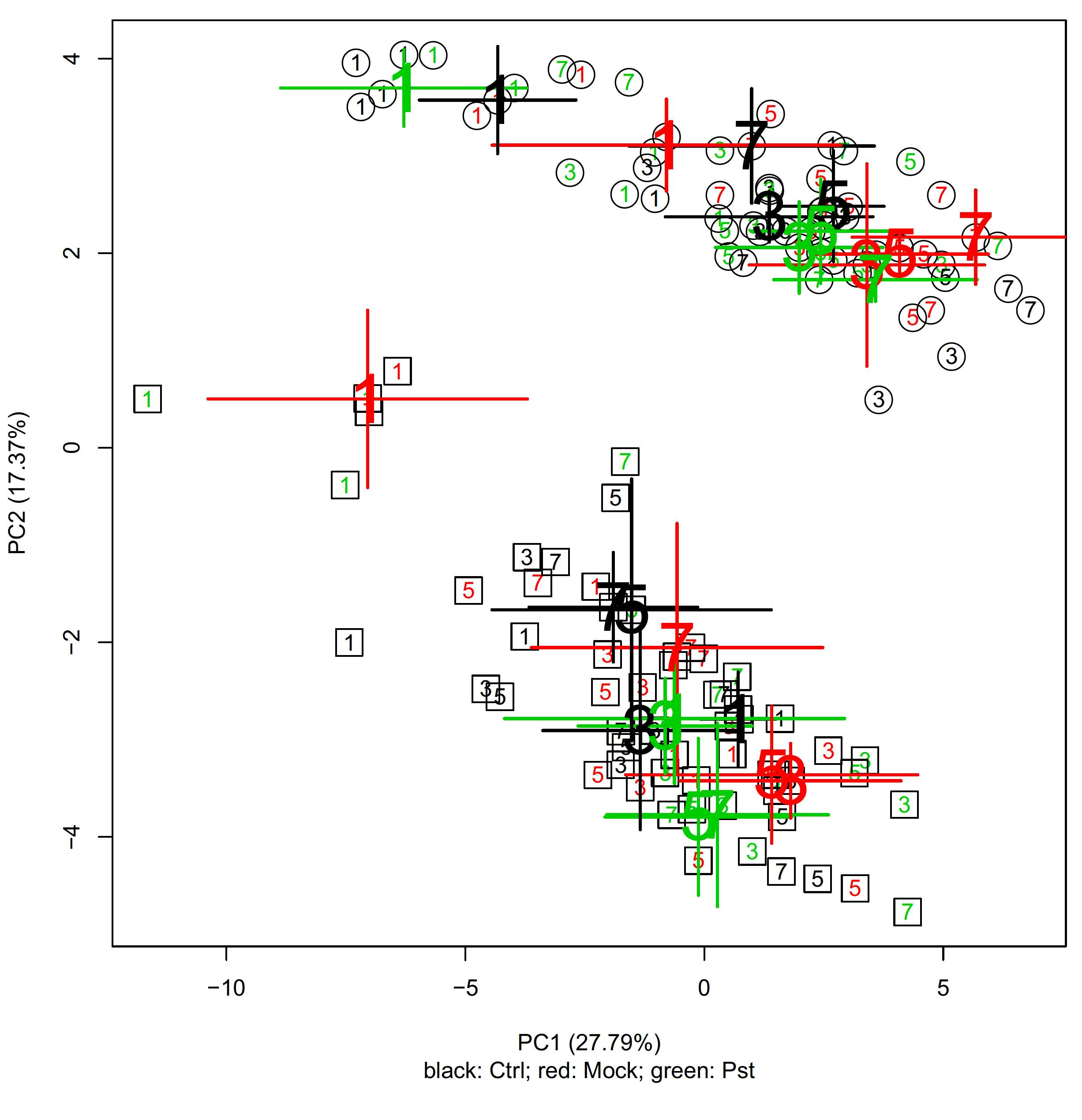

Supplement: Supplementary file 1 [file ijms-23-12087-s001.zip › SupFig S1_PstTS_PCA.png]

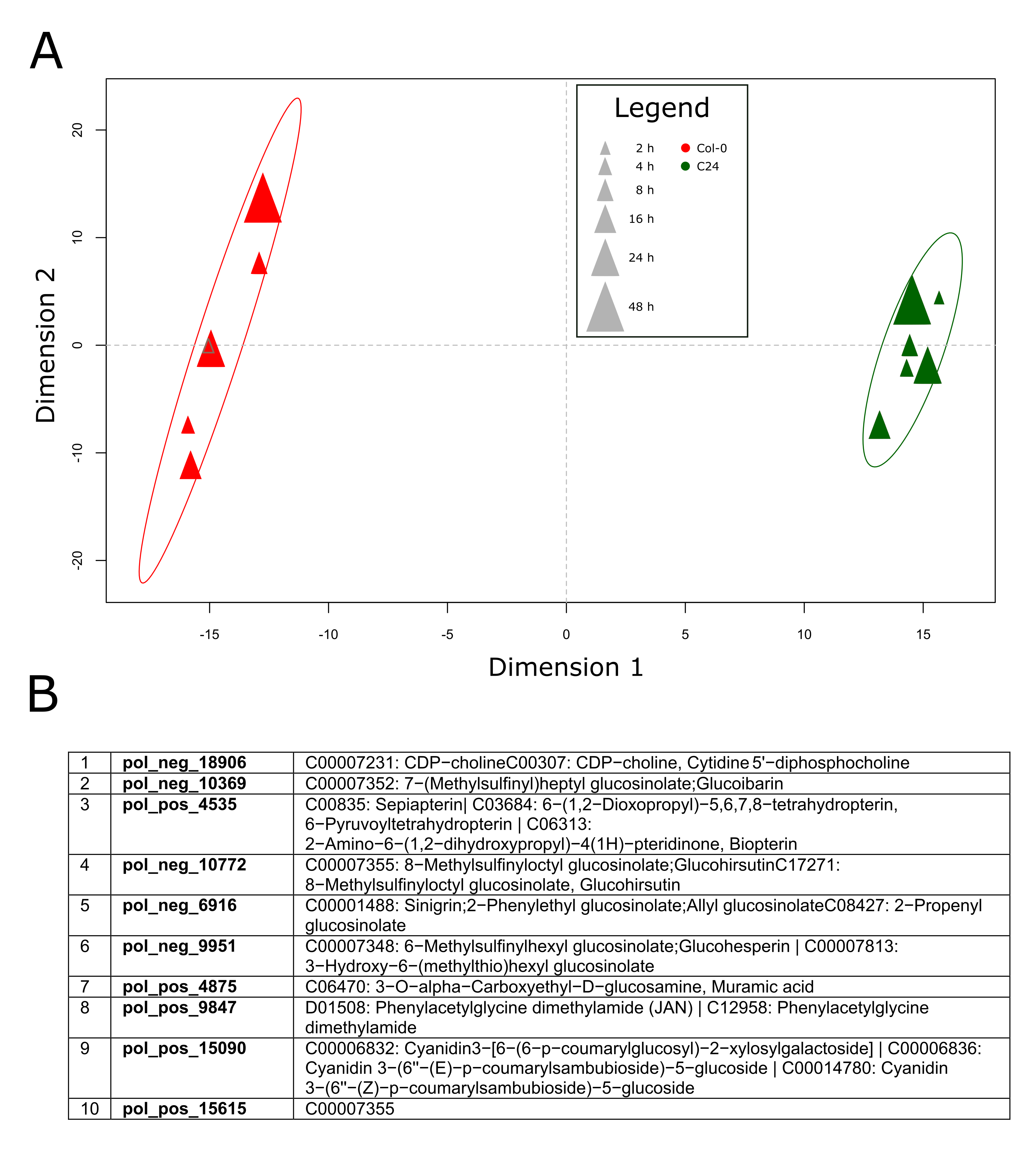

Supplement: Supplementary file 1 [file ijms-23-12087-s001.zip › SupFig S2_oplsda_gsl.png]

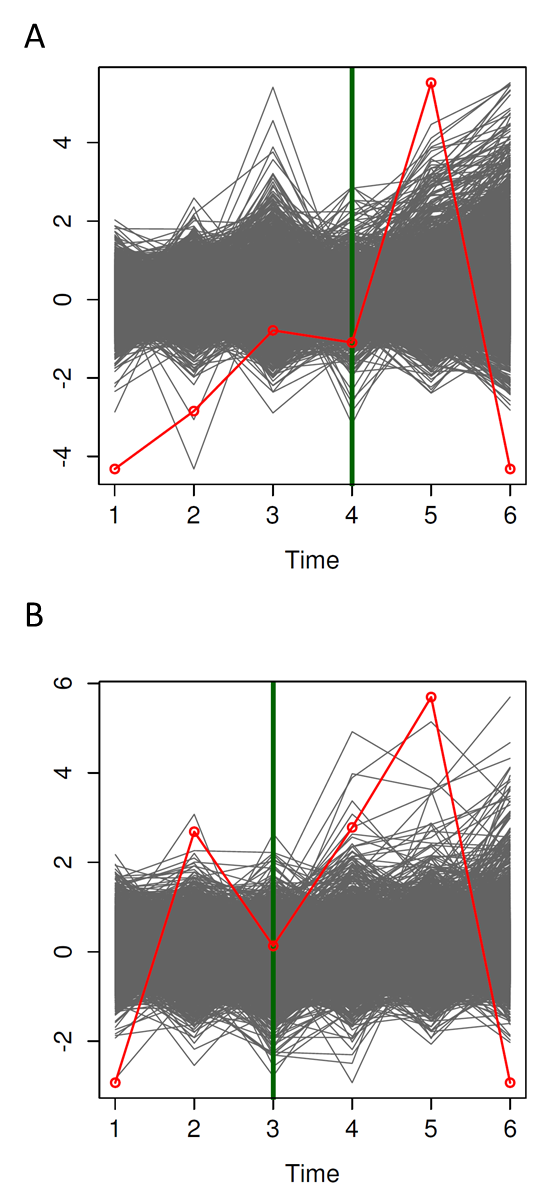

Supplement: Supplementary file 1 [file ijms-23-12087-s001.zip › SupFig S3_early-late.png]
